# Manufacturing and Assessing the New Orally Disintegrating Tablets, Containing Nimodipine-hydroxypropyl-β-cyclodextrin and Nimodipine-methyl-β-cyclodextrin Inclusion Complexes

**DOI:** 10.3390/molecules27062012

**Published:** 2022-03-21

**Authors:** Marian Novac, Adina Magdalena Musuc, Emma Adriana Ozon, Iulian Sarbu, Mirela Adriana Mitu, Adriana Rusu, Daniela Gheorghe, Simona Petrescu, Irina Atkinson, Dumitru Lupuliasa

**Affiliations:** 1 Department of Pharmaceutical Technology and Biopharmacy, Faculty of Pharmacy, “Carol Davila” University of Medicine and Pharmacy, 6 Traian Vuia Street, 020945 Bucharest, Romania; marian.novac@rez.umfcd.ro (M.N.); dumitru.lupuliasa@umfcd.ro (D.L.); 2 “Ilie Murgulescu” Institute of Physical Chemistry, 202 Spl. Independentei, 060021 Bucharest, Romania; arusu@icf.ro (A.R.); chiscan_danny@icf.ro (D.G.); simon_pet@icf.ro (S.P.); iatkinson@icf.ro (I.A.); 3 Department of Pharmaceutical Physics and Biophysics, Drug Industry and Pharmaceutical Biotechnologies, Faculty of Pharmacy, “Titu Maiorescu” University, 004051 Bucharest, Romania

**Keywords:** nimodipine, inclusion complexes, hydroxypropyl-β-cyclodextrin, methyl-β-cyclodextrin, orally disintegrating tablets

## Abstract

The aim of the present study was to manufacture new orally disintegrating tablets containing nimodipine–hydroxypropyl-β-cyclodextrin and nimodipine–methyl-β-cyclodextrin inclusion complexes. For obtaining a better quality of the manufactured tablets, three methods of the preparation of inclusion complexes, in a 1:1 molar ratio, were used comparatively; namely, a solid-state kneading method and two liquid state coprecipitation and lyophilization techniques. The physical and chemical properties of the obtained inclusion complexes, as well as their physical mixtures, were investigated using Fourier transformed infrared spectroscopy, scanning electron microscopy, X-ray diffraction analyses, and differential scanning calorimetry. The results showed that the lyophilization method can be successfully used for a better complexation. Finally, the formulation and precompression studies for tablets for oral dispersion, containing Nim–HP-β-CD and Nim–Me-β-CD inclusion complexes, were successfully assessed.

## 1. Introduction

In the last decade, great efforts have been focused on increasing the bioavailability and solubility of poorly soluble drugs. Generally, the developed techniques tried to enhance the drug dissolution rate by introducing the drug into a solid dispersion [1,2,3].

**Figure 1:**
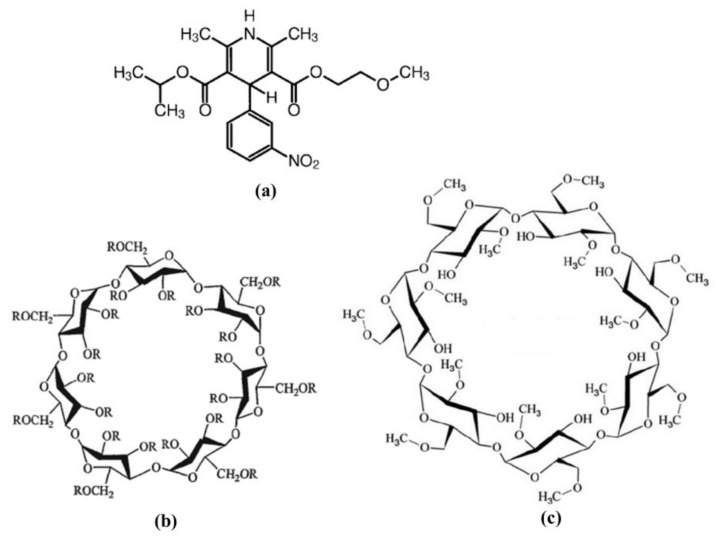
Chemical structures of (**a**) Nimodipine; (**b**) Hydroxypropyl-β-cyclodextrin (where R is H or isopropyl group); (**c**) Methyl-β-cyclodextrin.

Nimodipine (Nim), 3-(2-methoxyethyl) 5-propan-2-yl 2, 6-di-methyl-4-(3-nitrophenyl)-1, 4-dihydropyridine-3, 5-dicarboxylate (Figure 1a), is a 1,4-dihydropyridine calcium channel blocker that is used to reduce and treat the ischemic neurological diseases caused by ruptured congenital aneurysms [4,5,6]. Nimodipine is included in class II of the Biopharmaceutics Classification System (BSC), and consequently, it has low solubility and high permeability. Oral dosage forms of nimodipine have shown a good absorption at the gastrointestinal tract level, but due to its low systemic bioavailability, it has a limited clinical efficiency [7]. In addition to water insolubility, nimodipine easily decomposes when is exposed to light. One of the most effective methods to enhance its stability and solubility is to be included in the cavities of the cyclodextrins.

Cyclodextrins (Figure 1b-hydroxypropyl-β-cyclodextrin and Figure 1c-methyl-β-cyclodextrin) are cyclic (α-1, 4)-linked oligosaccharides, which are extensively used in the pharmaceutical field due to their environmentally friendly, low cost, hydrophilic, physical, chemical, and biological properties. Cyclodextrins were formed by the enzymatic degradation of starch. Due to their specific structure that is proper to form inclusion complexes, cyclodextrins can interact with different molecules both in solid-states and in solutions [8,9].

Considering the above-mentioned data, several studies reported the inclusion of nimodipine in different cyclodextrins cavities to obtain efficient orally administered dosage forms. The formulation of nimodipine, prepared by a direct compression technique with β-cyclodextrin complexes using various superdisintegrants, revealed fast dissolving tablets and better patient compliance [10].

The drug release behavior, in vitro, of nimodipine loaded hydroxypropyl-β-cyclodextrin based polymeric nanocapsules with a nanosized dimension prepared by the interfacial polyaddition of hydroxypropyl-β-cyclodextrin and isophorone diisocyanate in a miniemulsion system were affected by the diffusion of the drug from the polymer matrix [11].

The inclusion complexes of nimodipine with sulfobutyl ether-β-cyclodextrin (SBE-β-CD) and 2-hydroxypropyl-β-cyclodextrin (HP-β-CD) were prepared by different methods, such as kneading, co-evaporation, and freeze-drying at various stoichiometric ratios. The solubility and in vivo dissolution rate of nimodipine demonstrated that the complexes obtained by the kneading method showed the maximum dissolution rate [12]. 

Several studies tried to demonstrate the influence of the preparation method on the in vitro bioavailability of nimodipine [13,14]. The spray drying method showed greater solubility and drug dissolution rates of nimodipine-β-cyclodextrin and nimodipine-hydroxypropyl-β-cyclodextrin inclusion complexes, compared with the kneading method [15]. Nevertheless, the research contributions regarding the improvement of the solubility, the oral bioavailability, and the dissolution rate of nimodipine, as well as the physical and chemical properties of nimodipine inclusion complexes in cyclodextrins, still remain open.

Herein, the novelty of the present study is the manufacturing and evaluation of new orally disintegrating tablets containing nimodipine-hydroxypropyl-β-cyclodextrin and nimodipine-methyl-β-cyclodextrin inclusion complexes. The molecular structures of the two cyclodextrins used in this study are represented in Figure 1b—hydroxypropyl-β-cyclodextrin and Figure 1c—methyl-β-cyclodextrin. The two inclusion complexes (with a 1:1 molar ratio) were prepared by the solid-state kneading method, the liquid-state coprecipitation method, and the lyophilization method, and they were compared with their physical mixtures. The physical and chemical characterizations of the inclusion complexes were made by Fourier transform infrared spectroscopy (FTIR), X-ray diffraction analyses, scanning electron microscopy (SEM), and differential scanning calorimetry (DSC). Subsequently, the powders obtained by the lyophilization method were mixed with different excipients and subjected to preformulation studies to establish the best formulation and manufacturing processes for developing tablets of a suitable quality.

## 2. Materials and Methods

### 2.1. Materials 

Nimodipine (Nim) was purchased from Fagron, Greece; and hydroxypropyl-β-cyclodextrin (HP-β-CD) and methyl-β-cyclodextrin (Me-β-CD) were provided from the Global Holding Group Co., Ltd., (Ningbo, China). All used chemicals and solvents were of analytical reagent grade.

F-MELT^®^ was purchased from Fuji Chemical Industries Co., Ltd., Toyama, Japan; Disintequik™ ODT was purchased from Kerry Inc., Beloit, WI, USA; and PROSOLV^®^ SMCC HD 90 and EXPLOTAB^®^ were provided by JRS PHARMA GmbH & Co. KG, Rosenberg, Germany. LIGAMED^®^ MF-2-V, used in the study, was produced by Peter Graven NV, Venlo, Limburg, The Netherlands.

A Mettler Toledo AT261 balance (with 0.01 mg sensitivity) was used for weighing the raw materials.

### 2.2. Preparation of Binary Systems

Usually, when developing new inclusion complexes, the first step is to explore the CDs’ influence on the active ingredient solubility, according to the Higuchi and Connors phase solubility test [16]. As previous studies have been performed, in the present study, already published data was used. Semcheddine et al. determined that the aqueous solubility of nimodipine increased linearly as a function of the concentration of HP-β-CD, with a slope of <1, proving that this was due to the formation of a 1:1 molar ratio complex [12]. Moreover, Kopecký et al. investigated the stability constants of the inclusion complexes, nimodipine-cyclodextrins (including HP-β-CD and Me-β-CD), as well as the empirical linear equations for the calculation of the saturated nimodipine concentration at a given cyclodextrin concentration, establishing that 1:1 molar ratio is efficient in increasing nimodipine solubility [17].

Based on the previous literature results, nimodipine inclusion complexes with HP-β-CD and Me-β-CD in a minimal molar ratio of 1:1 were prepared. As the main purpose was to further incorporate the inclusion complexes in tablets for oral dispersion, firstly, a selection of the most suitable preparation methods for the inclusion compounds was made, meaning the technique that provides the total inclusion of nimodipine in the cavity of the CDs was essential to be established.

For this, both complexes were prepared by three different methods in both solid (kneading) and liquid states (coprecipitation and lyophilization) and was then subjected to the specific characterization and identification tests [18,19]. To obtain accurate results, the studies on the complexes were performed in comparison with the raw materials and with their simple physical mixtures prepared in the same 1:1 molar ratio. All binary systems were prepared maintaining the same amounts of the ingredients (1.0676 g of Nim and 3.9323 g of HP-β-CD for Nim–HP-β-CD system, and 1.2038 g of Nim and 3.7961 g of Me-β-CD for Nim–Me-β-CD system).

*Preparation of physical mixture between Nim and CDs*

The substances, weighed according to the molar ratio of 1:1, were mixed in a mortar for 15 min at room temperature until a homogeneous powder was obtained.

*Solid-state kneading method*

Nim and CDs (HP-β-CD and Me-β-CD) were weighed then transferred to a mortar and kneaded for 1 h with a low amount of methanol:water (50:50 *v/v*) solution. The wet adhesive mass that was obtained was passed through a 12-mesh sieve and the formed granules were dried at 30 °C for 24 h, then grounded until a fine powder was achieved.

*Liquid-state coprecipitation and lyophilization methods*

For both liquid-state methods, the mixture was prepared following the same steps.

The calculated amounts of Nim and CDs, according to the 1:1 molar ratio, were weighed, then Nim was dissolved in methanol and the CDs in water. The CDs solutions was added slowly to the Nim solution and then the mixture was stirred for 6 h at 700 rpm and at room temperature using a Heidolph MR 3001K magnetic stirrer.

In the case of the *coprecipitation* method, the obtained mixture was filtered, and the powder was dried at 25 °C in an exicator for 24 h.

For the *lyophilization* technique, the stirred mixture was frozen and then lyophilized at −60 °C for 12 h using the Christ ALPHA 1–2, B (Braun Biotech International, Germany) lyophilizer.

### 2.3. Binary Systems Characterization 

*Fourier Transform Infrared spectroscopy* (FTIR) measurements were performed using a NICOLET 6700 FT-IR spectrophotometer (Thermo Electron Corporation). FTIR spectra were obtained from the materials pressed into KBr pellets in the range of 400 to 4000 cm^−1^.

*Morphological studies* were carried out by Scanning Electron Microscopy (SEM) using an FEI Quanta 3D FEG (Brno, Czech Republic) model. Secondary electron images were recorded in high vacuum mode at an accelerating voltage between 5 and 20 kV on the dry samples placed on a double-adhesive carbon-coated tape and scanned without coating.

*Powder X-ray diffraction* (XRD) measurements were conducted on a Rigaku Ultima IV diffractometer using CuKα radiation (wavelength 1.5406 Å) in parallel beam geometry. The XRD patterns were recorded in a range between 10 and 70 with a speed of 2°/min and a step size of 0.02°.

DSC *measurements* of the samples were carried out on a differential scanning calorimetry power compensated DSC 8500 (Perkin–Elmer, Waltham, MA, USA) calorimeter with a cooling system (Intracooler III) at a heating rate of 10 °C min^−1^, using dry nitrogen purge gas with a flow rate of 20 mL min^−1^. Samples of ~1 mg were held at 25 °C for 2 min before heating from 25 °C to 250 °C. Sealed aluminum pans were used in the experiments for all the samples, and the temperature and heat flow rate scale of the DSC was performed using high-purity indium as a standard (*T*_fus_ = 156.7 °C and Δ*H*_fus_ = 28.5 J g^−1^). The Pyris Software for Windows was used to calculate the thermal effects. Thermograms were normalized to the sample weight.

### 2.4. Formulation and Precompression Studies for Tablets for Oral Dispersion Containing Nim–HP-β-CD and Nim–Me-β-CD Inclusion Complexes

Considering the results of the complexes’ physical and chemical characterization, the nimodipine inclusion was at its maximum when using the lyophilization method for preparation, meaning these systems will show a better in vivo performance. 

As the goal of this research was to obtain tablets for oral dispersion, the ideal manufacturing method is by direct compression, due to all its advantages, but mainly due to the fact that no humidity is involved in the process and the ingredients are well protected in this way. To ensure that final tablets will present adequate pharmaco-technical and stability characteristics, it is important to prepare a direct compression blend with high flowability and compressibility attributes. 

*Powder for direct compression formulation*

The amounts of inclusion complexes and excipients are calculated, and the dosage of nimodipine for one tablet was established to be 30 mg.

The chosen formulations, F1-F3 containing the Nim–HP-β-CD inclusion complex and F4-F6 containing Nim–Me-β-CD, are as follows (Table 1):

Prosolv^®^ SMCC HD 90, the grade of silicified microcrystalline cellulose, has the properties to highly increase the compaction and flow properties of the formulation to eliminate the use of glidants, to reduce bridging, to enhance consolidation, and to improve content uniformity, as silicification provides flow that is comparable to doubling the particle size of MCC.

F-Melt^®^ is a spray-dried excipient used in orally disintegrating tablets that contain saccharides, disintegrating agents, and inorganic excipients, it exhibits excellent tableting properties and facilitates rapid water-penetration for a fast disintegration time [20,21].

Disintequik™ ODT is a co-processed excipient, consisting of mannitol, monohydrate lactose, dextrose monohydrate, and crospovidone, designed for the direct compression process for use in orally disintegrating tablets. It can be blended with a flavor, an active suitable lubricant, and an additional sweetener if desired, and then it can be directly compressed into tablets.

Explotab^®^, containing sodium starch glycolate, is the sodium salt of cross-linked carboxymethyl starch that acts as a superdisintegrant through rapid swelling due to the adsorption of large amounts of water, leading to faster disintegration. Moreover, due to its spherical shape, it has good flow properties.

Magnesium stearate is used for its lubricant properties. Ligamed^®^ MF-2-V has a high specific surface area and the fine particles offer a high releasing speed during tablet pressing, as well as the consistent physical performance of the tablets, such as hardness and dissolution profiles. It is also used as a flowability agent.

*Powder for direct compression preparation*

The ingredients were passed through a 20-mesh sieve, then accurately weighed and mixed in a CMP 12 Plexiglas cube blender, produced by Pharmag GmbH, Klipphausen, Germany, at a speed of 30 rpm, for 30 min, at room temperature. 

*Powder for the direct compression physical properties evaluation*

*Fineness*: This was estimated by analytical sieving, using a CISA Sieve Shaker Mod. RP 10, produced by CisaCedaceria Industrial, Spain. Fifty grams of each bulk powder were passed by through a vibrating standard sieve set arranged with the coarsest at the top and the finest at the bottom, shaken for 20 min. Then, the sample retained on each sieve was collected and weighed.

*Flowability*: This was analyzed by determining the angle of repose, the flowing time, and the flowing and rate, registered by 60 g samples of each powder when passing through an orifice with a standardized diameter. An Automated Powder and Granulate Testing System PTG-S3, fabricated by Pharma Test Apparatebau GmbH, Germany, was used for this study. The determinations were performed five times for each powder.

*Compressibility*: This was performed with a Vankel Tap Density Tester, produced by Vankel Industries Inc., Santa Clara, CA, USA. The bulk and tapped density, the Hausner ratio (HR), and the Carr Index (*CI*) were calculated. Fifty grams of each powder were gently introduced into the graduated cylinder and the bulk volume was measured. Then, it was subjected to a defined number of mechanical shocks, measuring the tapped volume of the material. HR and *CI* were used to predict the propensity of the powder to be compressed, where HR is the ratio between tapped density and bulk density and the *CI* is calculated according to the Equation (1):

(1) $$CI\left(\%\right)=100\times \frac{\left(\rho_{tapped}-\rho_{bulk}\right)}{\rho_{tapped}}$$

where *ρ_tapped_* is the tapped bulk density of the material (kg/m^3^) and *ρ_bulk_* is the loose bulk density of the material (kg/m^3^).

An HR value under 1.25 means that the powder is freely flowing, and a *CI* value lower than 10 indicates an excellent flowability and compressibility of the material [22].

*Moisture content* was determined by thermogravimetric method, measuring the powder loss on drying, with an HR 73 Mettler Toledo halogen humidity analyzer from Mettler-Toledo GmbH, Greifensee, Switzerland.

*Formulation of the tablets containing Nim–HP-β-CD and Nim–Me-β-CD inclusion complexes*

The preformulation studies proved that F1 and F4 bulk materials have suitable attributes to be processed by the direct compression method; therefore, the final formulation of the tablets is presented in Table 2. For manufacturing technology, it was decided to compress the materials at different tableting forces (a medium one—30 N and a high one—60 N) to establish which of them leads to orodispersible tablets of higher quality. In this way, F7 represents F1 compressed at 30 N, F8 is F1 at 60 N, F9 is F4 at 30 N, and F10 represents F4 at 60 N.

### 2.5. The Manufacturing of Orally Disintegrating Tablets

The direct compression was realized in a single-post eccentric machine Korsch EK-O type, equipped with 12 mm flat punches, and it was adjusted to obtain tablets with an average mass of 500 mg, corresponding to a content of 30 mg nimodipine/orodispersible tablets. Two different compression forces, 30 N and 60 N, were applied. All the series of tablets for oral dispersion were analyzed using European Pharmacopoeia recommendations [23] to establish the best formulation and manufacturing process for obtaining tablets of a suitable quality. 

### 2.6. Quality Characterization of the Orodispersible Tablets 

*Organoleptic evaluation*

The general appearance and color of the tablets were evaluated according to compendial specifications [23]. 

*Dimensions (diameter and thickness)*

The diameter and the thickness were determined on ten tablets of each formulation with a VK 200 Tablet Hardness Tester, produced by Vanderkamp, Edison, NJ, USA.

*Weight uniformity*

For determining the weight uniformity, 20 tablets of each formulation were individually weighed, and the average mass was calculated. The maximum percentage difference allowed is 5% [23]. 

*Hardness*

Hardness was determined with a VK 200 Tablet Hardness Tester. Ten tablets of each batch were placed between the two anvils and the load required to crush them was recorded. 

*Friability*

Ten tablets of each formulation were accurately weighed and placed in the Vankel friabilator, rotating at 25 rpm for 5 min. Then, the tablets were de-dusted and reweighed. The loss in weight is calculated and expressed as a percentage. The acceptable values are not more than 1.0%. 

### 2.7. In Vitro Disintegration Time 

The test was performed on six tablets of each batch, using a Erweka DT 3 apparatus, produced by Erweka^®^ GmbH, Germany. The disintegration performance was determined in two different media: distilled water at 37 ± 0.5 °C, according to compendial standards [23], and a simulated saliva phosphate buffer with a pH of 6.8 at 37 ± 2 °C. The time needed for complete disintegration, with no residual mass left on the screen, was measured in seconds.

### 2.8. In Vitro Dissolution Studies

The dissolution studies were carried out using the USP basket apparatus (ERWEKA DT800 HH model, Heusenstamm, Germany) at a rotation speed of 50 rpm. The test was performed on two different dissolution media: the compendial dissolution medium (900 mL of 0.01-N HCl) [USP] and a more biorelevant medium simulating the composition of the saliva containing 8-g/L NaCl, 0.19-g/L KH_2_PO_4_, and 2.38-g/L Na_2_HPO_4_ with a pH of 6.8 [24]. The temperature was maintained at 37 ± 0.5 °C and after 30 min, 2 mL samples were withdrawn and the amount of nimodipine dissolved was assessed by the UV–VIS spectrophotometric method (UV-VIS Nicolet Evolution 100 spectrometer) at the absorption maximum of 239 nm. The determinations were made on six tablets of each batch.

## 3. Results and Discussion

**Figure 2:**
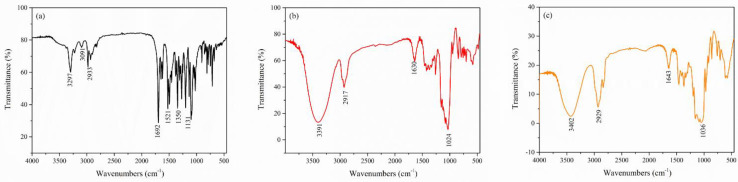
FTIR spectra of (**a**) Nim; (**b**) HP-β-CD; (**c**) Me-β-CD.

*FTIR analysis:* FTIR spectroscopy was carried out to evaluate the nimodipine and cyclodextrin interactions in the physical mixture, solid-state, and liquid-state preparation methods. The FTIR spectra of pure compounds (Nim, HP-β-CD and Me-β-CD) were shown in Figure 2a–c. The FTIR spectra of nimodipine from Figure 2a showed the presence of its main characteristic peaks at 3297 cm^−1^ due to the N–H stretching vibration, at 3091 cm^−1^ assigned to the C–H aromatic stretching vibration, at 2933 cm^−1^ due to the C–H aliphatic stretching vibration, at 1692 cm^−1^ due to the C=O stretching vibration in ester, at 1641 cm^−1^ due to the C=C stretching vibration, at 1619 cm^−1^ due to the C=C aromatic ring, at 1521 cm^−1^ attributed to the −NO_2_ vibration, at 1350 cm^−1^ due to the −C–CH_3_ bond, and at 1131 cm^−1^ due to the −C–O ester vibration [12]. The FTIR spectra of HP-β-CD (Figure 2b) and Me-β-CD (Figure 2c) showed the main characteristic peaks corresponding to polysaccharides, namely, the peak at 3391 cm^−1^ for HP-β-CD and 3402 cm^−1^ for Me-β-CD due to the O–H stretching vibration, at 2917 cm^−1^ for HP-β-CD and 2929 cm^−1^ for Me-β-CD due to the C–H stretching vibration, at 1630 cm^−1^ for HP-β-CD and 1643 cm^−1^ for Me-β-CD due to the H–O–H bending vibration, and at 1024 cm^−1^ for HP-β-CD and 1036 cm^−1^ for Me-β-CD due to the C–O bond vibration [25,26].

**Figure 3:**
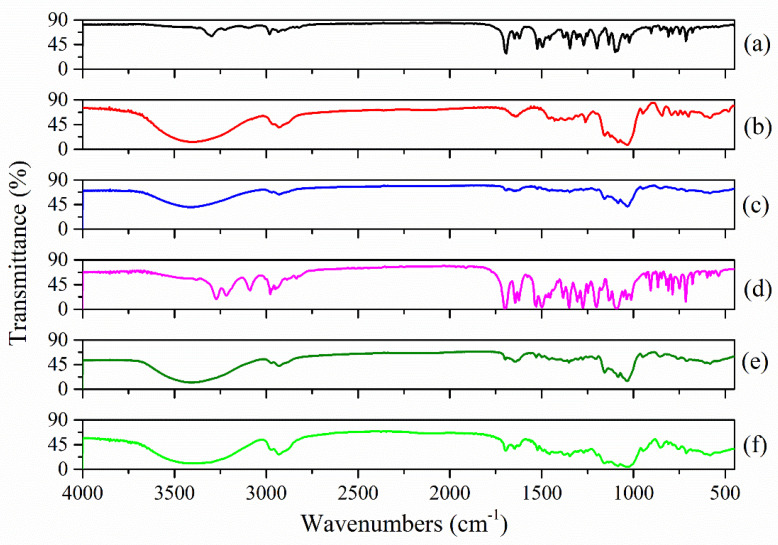
FTIR spectra of (**a**) Nim; (**b**) HP-β-CD; (**c**) Nim–HP-β-CD physical mixture; (**d**) Nim–HP-β-CD coprecipitation method; (**e**) Nim–HP-β-CD lyophilization method; (**f**) Nim–HP-β-CD kneading method.

Figure 3 shows the FTIR spectra recorded for the (a) Nim; (b) HP-β-CD; (c) Nim–HP-β-CD physical mixture; (d) Nim–HP-β-CD coprecipitation method; (e) Nim–HP-β-CD lyophilization method; and the (f) Nim–HP-β-CD kneading method. The FTIR spectra of the Nim–HP-β-CD physical mixture (Figure 3c) and the inclusion complex obtained from the lyophilization method (Figure 3e) are comparable, but the peak intensities are more intense in the case of the lyophilization process. The characteristic peaks of nimodipine nearly disappeared or were shifted to a higher frequency, and only the peaks of HP-β-CD were observed at approximatively the same wavenumber. Thus, one can conclude that some characteristic functional groups from the drug molecule were involved in the inclusion processes. This demonstrates that the drug was included in the HP-β-CD cavity, and even a partial inclusion was observed in the case of the physical mixture. The FTIR spectrum of the Nim–HP-β-CD inclusion complex formed by the coprecipitation method (Figure 3d) demonstrated that almost all the peaks of nimodipine and HP-β-CD occurred without any significant differences, proving that in this case, only a partial inclusion occurred for a 1:1 molar ratio. In the case of the inclusion complex obtained using the kneading method (Figure 3f), the FTIR spectrum demonstrated some changes compared with previous spectra. Several peaks attributed to nimodipine were still present in the inclusion compound spectrum, which demonstrates that the inclusion process was not complete. The characteristic C=O absorption band of nimodipine, in the case of the lyophilization process (Figure 3e) shifted to a higher frequency (1701 cm^−1^) and was attributed to the disruption of nimodipine’s strong hydrogen bonds [27]. 

**Figure 4:**
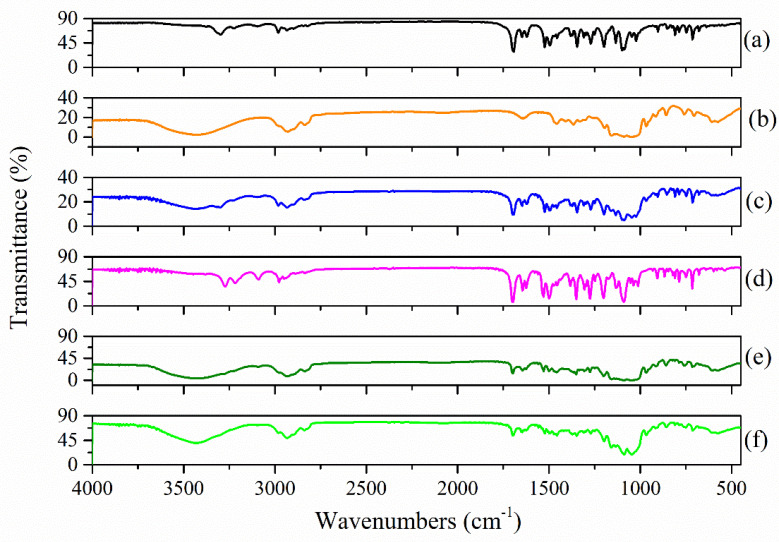
FTIR spectra of (**a**) Nim; (**b**) Me-β-CD; (**c**) Nim–Me-β-CD physical mixture; (**d**) Nim–Me-β-CD coprecipitation method; (**e**) Nim–Me-β-CD lyophilization method; (**f**) Nim–Me-β-CD kneading method.

Figure 4 shows the FTIR spectra recorded for the (a) Nim; (b) Me-β-CD; (c) Nim–Me-β-CD physical mixture; (d) Nim–Me-β-CD coprecipitation method; (e) Nim–Me-β-CD lyophilization method; and the (f) Nim–Me-β-CD kneading method. 

There are significant differences in the FTIR spectra of the Nim–Me-β-CD inclusion complexes obtained by the three methods of preparation, compared with Nim–HP-β-CD. The Nim–Me-β-CD physical mixture (Figure 4c) contains all the absorption peaks corresponding to nimodipine and Me-β-CD, demonstrating no interaction between the two compounds for this case. The FTIR spectrum of the compound obtained by the coprecipitation method (Figure 4d) contains almost all the vibration bands of Nim, and that demonstrates there is no complete inclusion process for a 1:1 molar ratio, whereas both lyophilization (Figure 4e) and kneading methods (Figure 4f) revealed the formation of new chemical bonds between Nim and Me-β-CD [28]. Moreover, in this case, the inclusion process is completed using the lyophilization technique. 

**Figure 5:**
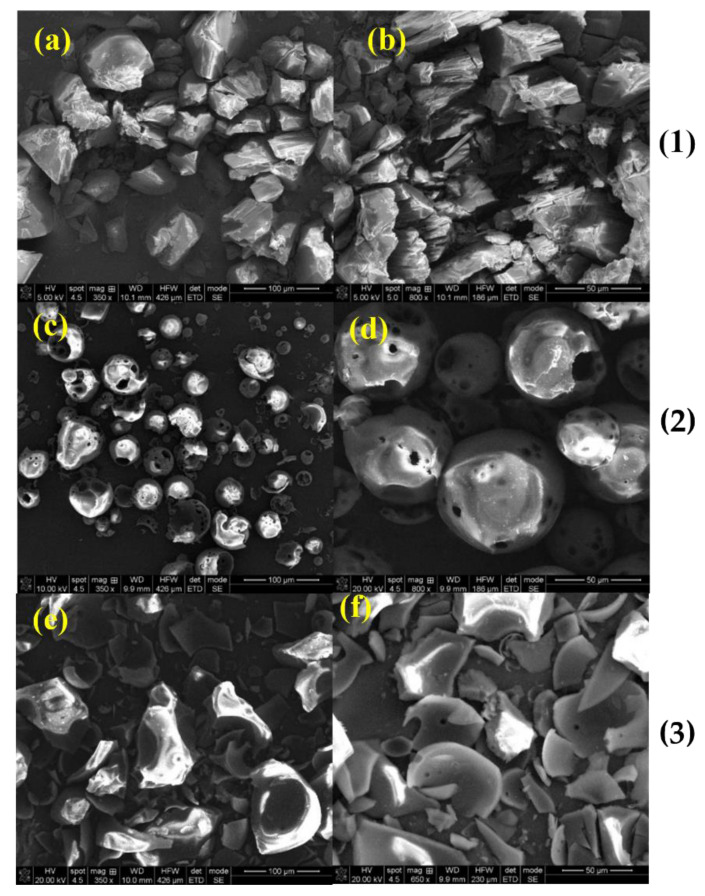
SEM images, at different magnifications, of (**1**) Nim; (**2**) HP-β-CD; and (**3**) Me-β-CD; (**a**) 350×; (**b**) 800×; (**c**) 350×; (**d**) 800×; (**e**) 350×; (**f**) 650×.

**Figure 6:**
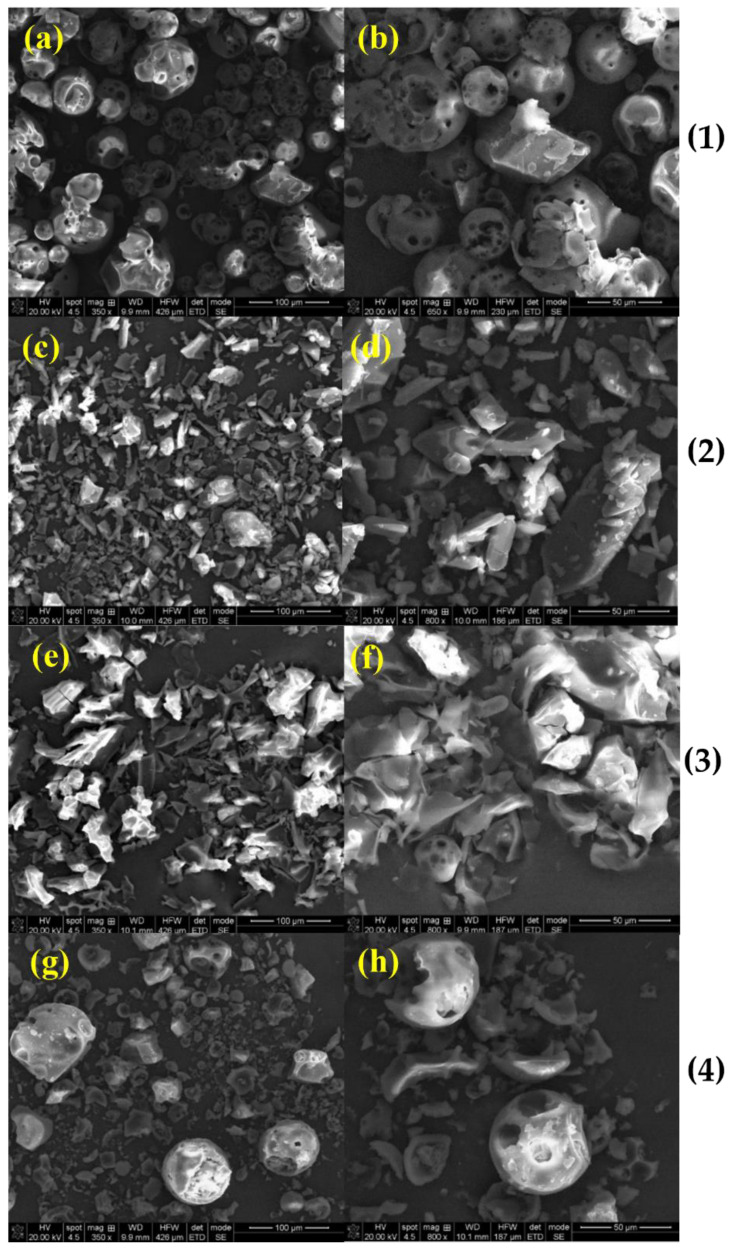
SEM images, at different magnifications, of (**1**) Nim–HP-β-CD physical mixture; (**2**) Nim–HP-β-CD coprecipitation method; (**3**) Nim–HP-β-CD lyophilization method; and (**4**) Nim–HP-β-CD kneading method; (**a**) 350×; (**b**) 650×; (**c**) 350×; (**d**) 800×; (**e**) 350×; (**f**) 800×; (**g**) 350×; (**h**) 800×.

**Figure 7:**
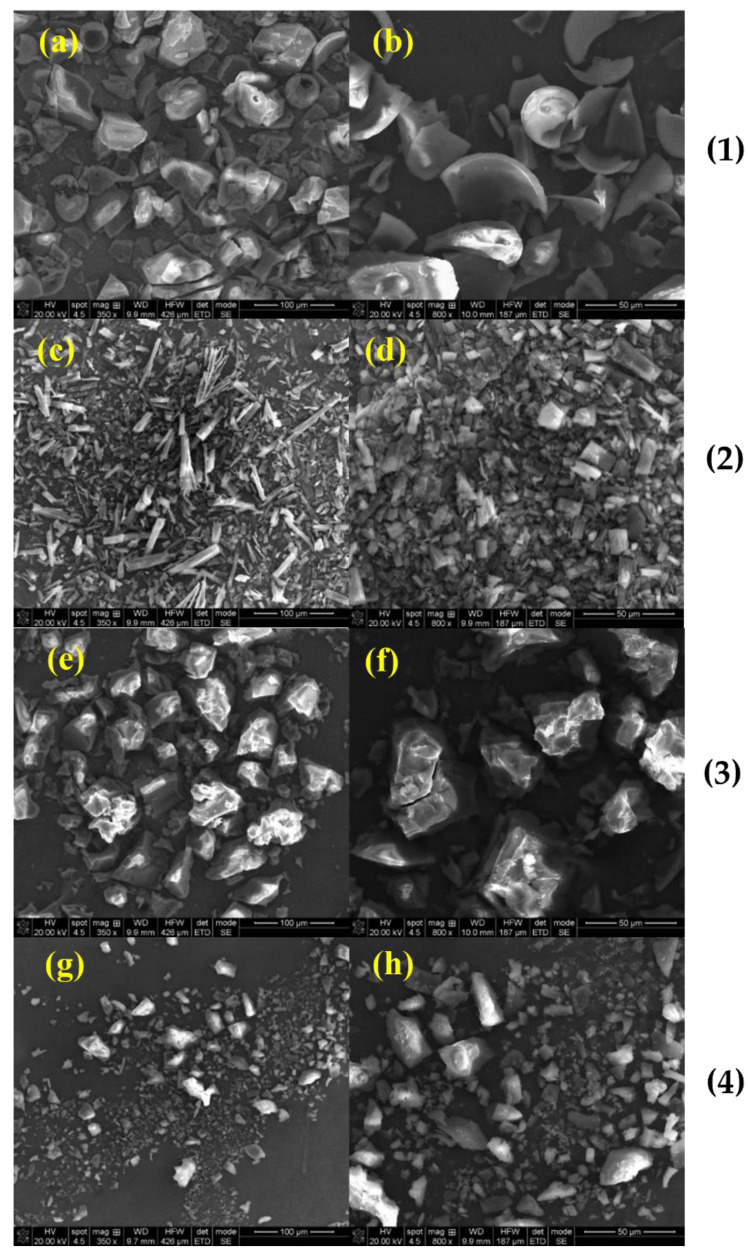
SEM images, at different magnifications, of (**1**) Nim–Me-β-CD physical mixture; (**2**) Nim–Me-β-CD coprecipitation method; (**3**) Nim–Me-β-CD lyophilization method; and (**4**) Nim–Me-β-CD kneading method; (**a**) 350×; (**b**) 800×; (**c**) 350×; (**d**) 800×; (**e**) 350×; (**f**) 800×; (**g**) 350×; (**h**) 800×.

*SEM analysis:* SEM is a qualitative method used to study the structural aspect of the materials. In the present study, it was performed in order to evaluate the microscopic morphology from the surface of the complexes and their approximate dimensions. The SEM images provided useful details regarding the structural changes that occurred in the complexes in comparison to the raw materials and their simple physical mixtures, due to the inclusion process. The morphologies of pure compounds are presented in Figure 5. The Nim–HP-β-CD and Nim–Me-β-CD inclusion complexes and physical mixtures are presented in Figure 6 and Figure 7, respectively.

Large irregular particles of commercial nimodipine (Nim) are depictable in Figure 5 [29], while spherical shells with circular holes of hydroxypropyl-β-cyclodextrin, (Hp-β-CD), with a particle ranging from 25 to 100 μm, are shown in Figure 5 [30]. For methyl-β-cyclodextrin, Me-β-CD, fragments of spherical shells are presented in Figure 5 [31].

Different morphologies were obtained according to the preparation methods, e.g., the physical mixture, coprecipitation, lyophilization, and kneading methods.

Thus, the Nim–HP-β-CD physical mixture shows some broken cyclodextrin shells and a smaller amount of nimodipine that has filled the cyclodextrin voids (Figure 6). The coprecipitation derived sample gives evidence of the cyclodextrin shell shards and smaller nimodipine particles (Figure 6). In Figure 6, for the nimodipine with HP-β-CD sample obtained by lyophilization, the SEM micrographs point out a heterogeneous mixture between the two components. Cyclodextrin shells in the kneading sample seem to be filled with nimodipine, whereas smaller nimodipine particles and cyclodextrin fragments are also present in this sample (Figure 6). Among the four methods of preparation, the kneading method caused the formation of small particles of nimodipine, as well as clogged cyclodextrin shells, but the lyophilization method provided a good complexation. The SEM images clearly indicated that the Nim crystallinity was highly reduced in lyophilization complexes compared to the other methods of preparation.

No morphology modification was observed (Figure 7) for the methyl-β-cyclodextrin-based materials obtained by the physical blending of the components. Conversely, the coprecipitation method yielded a bench of acicular-shaped nimodipine and powdered methyl-β-cyclodextrin (Figure 7). After the lyophilization process, the SEM images of Nim–Me-β-CD, from Figure 7, showed large aggregates. The finest nimodipine particles were noticed along with small broken cyclodextrin fragments in the sample obtained using the kneading method, in Figure 7. The SEM analysis is not conclusive regarding the forming of an inclusion complex, but it is fruitful to demonstrate the sample homogeneity and crystallinity.

**Figure 8:**
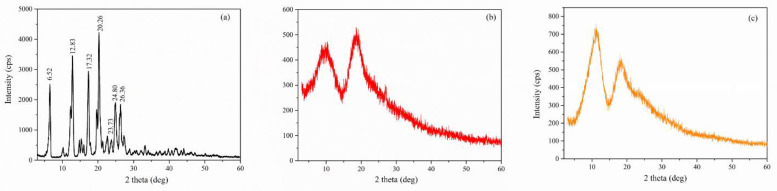
XRD pattern of (**a**) Nim (mod I); (**b**) HP-β-CD; (**c**) Me-β-CD.

*X-ray diffraction:* The XRD diffractograms of pure compounds (Nim, HP-β-CD, and Me-β-CD) are displayed in Figure 8a–c. The XRD pattern of Nim (Figure 8a) exhibited a series of well-defined and strong diffraction peaks with maxima at 2θ = 6.52°, 12.83°, 17.32°, 20.26°, 23.73°, 24.80°, and 26.36°, which indicated its crystalline nature, according to the JCPDS card 00-410-7268. The XRD pattern of the Nim used in this study corresponds to that of the Nim crystal modification I (mod I) which is the racemic compound. Similar XRD patterns were also evidenced in the literature [12,32,33]. Both pure HP-β-CD (Figure 8b) and Me-β-CD (Figure 8c) were found to be in the amorphous phase, as evidenced by the appearance of a broad peak at around 2θ = 9.7° and 18.6° for HP-β-CD, and at around 2θ = 11.2° and 18.3° for Me-β-CD. 

**Figure 9:**
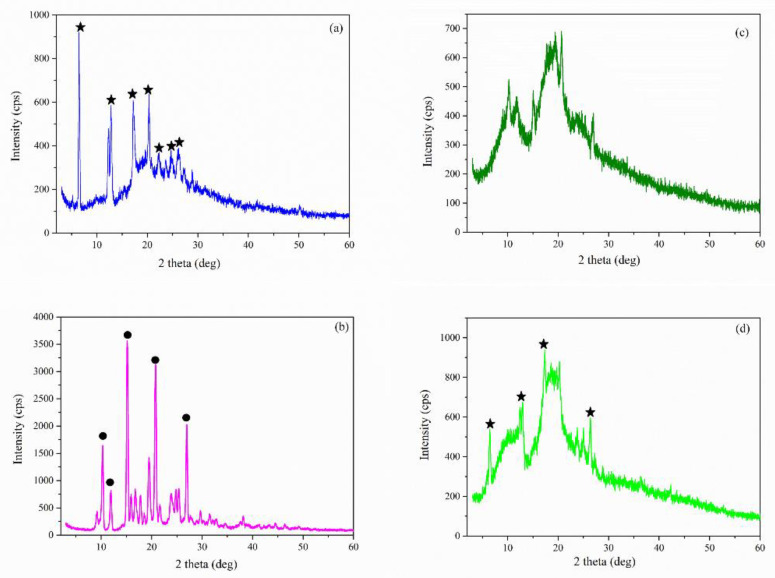
XRD pattern of (**a**) Nim–HP-β-CD physical mixture; (**b**) Nim–HP-β-CD coprecipitation method; (**c**) Nim–HP-β-CD lyophilization method; (**d**) Nim–HP-β-CD kneading method (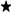
—Nim mod I; 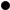
—Nim mod II).

Figure 9 shows the XRD pattern recorded for the (a) Nim–HP-β-CD physical mixture; (b) Nim–HP-β-CD coprecipitation method; (c) Nim–HP-β-CD lyophilization method; and the (d) Nim–HP-β-CD kneading method. Even though Figure 9a displays a reduction in the nimodipine peak intensities, the characteristic diffraction peaks of Nim mod I were evidenced in the physical mixture. The XRD pattern of the Nim–HP-β-CD physical mixture (Figure 9a) confirms a partial interaction between nimodipine and HP-β-CD, even though a simple mixture between the two components. Regarding the partial inclusion of the drug in the physical mixture of NM-HP-β-CD, it was due to the good affinity of Nim to form the inclusion complex with HP-β-CD, even in the physical mixing procedure using a 1:1 molar ratio. The XRD pattern obtained for the inclusion complex formed the by coprecipitation method (Figure 9b) showed a polymorphic change in nimodipine. The XRD pattern from Figure 9b showed the presence of the Nim modification II (mod II) which is the conglomerate form (2θ = 10.2°, 11.8°, 15.1°, 20.6°, 26.8°) [33]. In the case of the lyophilization (Figure 9c) and kneading (Figure 9d) methods, the disappearance of the characteristic peaks of nimodipine mod I sustain the conclusion of a stronger interaction and the formation of the inclusion complex, denoted in the case of the lyophilization technique. In the XRD pattern of the sample obtained by the kneading method (Figure 9d), some peak diffractions of Nim are superposed on the amorphous pattern of the HP-β-CD, which indicates a partial inclusion.

**Figure 10:**
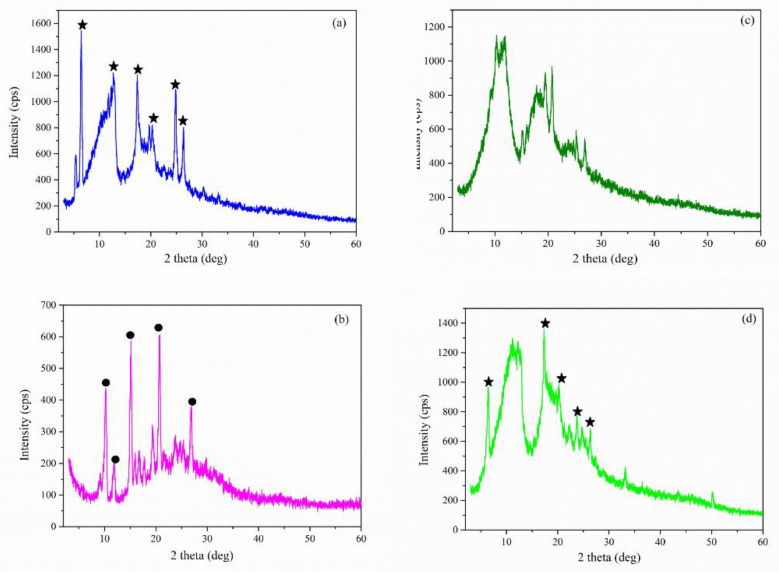
XRD pattern of (**a**) Nim–Me-β-CD physical mixture; (**b**) Nim–Me-β-CD coprecipitation method; (**c**) Nim–Me-β-CD lyophilization method; (**d**) Nim–Me-β-CD kneading method (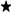
—Nim mod I; 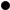
—Nim mod II).

Figure 10 displays the XRD pattern recorded for the Nim–Me-β-CD physical mixture, Nim–Me-β-CD coprecipitation method, Nim–Me-β-CD lyophilization method, and Nim–Me-β-CD kneading method.

Similar to the case of the Nim–HP-β-CD inclusion complexes, the XRD diffractograms of Nim–Me-β-CD were obtained using different synthesis methods from Figure 10a–d, which proved the partial or total inclusion of Nim in the Me-β-CD cavity. The visible decrease in the nimodipine peak intensities in the case of lyophilization (Figure 10c) and kneading (Figure 10d) methods, even their disappearance, indicates that an inclusion complex is formed [33]. The coprecipitation method favors the polymorphic change from mod I to mod II of nimodipine, as highlighted in Figure 10b. The evidence of the Nim–Me-β-CD inclusion complex formation is more obvious for the lyophilization method, confirming the FTIR findings.

**Figure 11:**
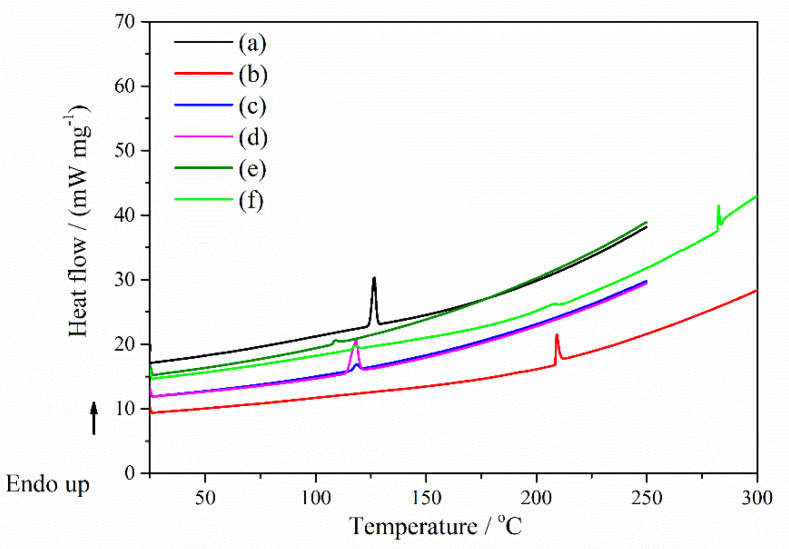
DSC curves of (**a**) Nim; (**b**) HP-β-CD; (**c**) Nim–HP-β-CD physical mixture; (**d**) Nim–HP-β-CD coprecipitation method; (**e**) Nim–HP-β-CD lyophilization method; (**f**) Nim–HP-β-CD kneading method.

*DSC measurements:* Concerning the DSC result of the Nim pure compounds (Figure 11a), the thermal curves showed the typical feature of a crystalline and pure compound in the case of nimodipine with a sharp endothermal peak corresponding to its melting process (peak temperature of *T*_m_ = 126.48 °C), characteristic for mod I of the nimodipine form [34]. Figure 11 showed the DSC curves of Nim, HP-β-CD, Nim–HP-β-CD physical mixture, Nim–HP-β-CD coprecipitation method, Nim–HP-β-CD lyophilization method and Nim–HP-β-CD kneading method. While the melting peak of nimodipine almost disappeared in the case of the lyophilization and kneading methods (Figure 11e,f), it was still visible in the physical mixture and was more evidenced in the coprecipitation method, but shifted to lower temperatures. The shifting of the endothermal peak of Nim to a lower temperature indicates some type of interaction between the drug and HP-β-CD. It can be supposed that the physical mixtures and the compound formed by the coprecipitation method of Nim with HP-β-CD certainly contained some amount of crystalline Nim, but the lyophilization and kneading sample processes probably contained it in small quantities or did not contain any crystalline nimodipine. 

**Figure 12:**
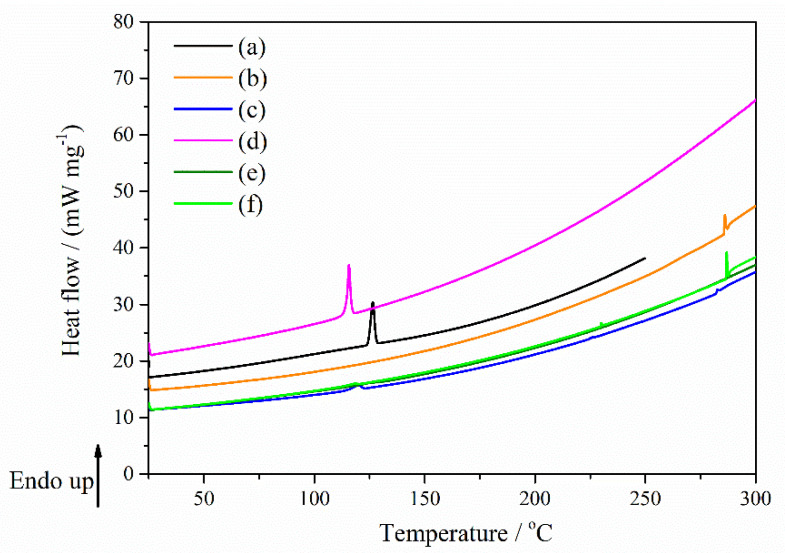
DSC curves of (**a**) Nim; (**b**) Me-β-CD; (**c**) Nim–Me-β-CD physical mixture; (**d**) Nim–Me-β-CD coprecipitation method; (**e**) Nim–Me-β-CD lyophilization method; (**f**) Nim–Me-β-CD kneading method.

Figure 12 displays the DSC curves of Nim, Me-β-CD, Nim–Me-β-CD physical mixture, Nim–Me-β-CD coprecipitation method, Nim–Me-β-CD lyophilization method and Nim–Me-β-CD kneading method. The melting point of nimodipine is still observed with a higher intensity in the coprecipitation method (Figure 12d), proving that the inclusion complex was not completely formed using this preparation technique in the case of nimodipine with Me-β-CD. The Nim–Me-β-CD physical mixture (Figure 12c) showed a small peak that shifted to a lower temperature, proving that some interactions between the drug and Me-β-CD occur, even in this case. 

The absence of the melting endothermic peak at 126.48 °C can be noticed for the lyophilization (Figure 12e) and kneading (Figure 12f) working procedures, providing some evidence of total complexation, as the melting point of the drug tends to disappear when it is embedded inside the cyclodextrin cavity [35]. An explanation may be due to the totally entrapping of the nimodipine in the Me-β-CD cavity. The disappearance of the melting endotherm is evidence that in these systems may cause a reduction in drug crystallinity, or probably a partial dispersion at a molecular level in the solid product. These findings confirm that the lyophilization and kneading methods were adequate methods for the successful preparation of nimodipine inclusion complexes in a 1:1 molar ratio. The DSC data are supported by those obtained from FTIR, X-ray diffraction, and SEM analyses.

The thermal DSC data and their characteristic thermodynamic parameters were summarized in Table 3.

Based on the DSC results, it was observed that the lyophilization method is the best way to obtain inclusion complexes of nimodipine with HP-β-CD and Me-β-CD, respectively, in a 1:1 molar ratio. As evident from the DSC curves in Figure 11 and Figure 12, the coprecipitation and kneading processes yield a partial nimodipine inclusion complex in the case of both cyclodextrins. 

*Preparation of binary systems*

The prepared binary systems were all white, homogenous, fine powders, with different appearances depending on the manufacturing method.

*The formulation and precompression studies for tablets for oral dispersion containing Nim–HP-β-CD and Nim–Me-β-CD inclusion complexes.*

Usually, the lyophilized powders display low flowability and compressibility properties, and are not suitable for incorporation in tablets and for direct compression technology. To meet this challenge, accurate formulation and process parameters are needed.

The selection of the excipients is a critical step in the formulation; therefore, for the present study, different ingredients in various amounts were used to determine the precompression parameters [20,36]. Based on the obtained results, the final formulation will be decided.

The assessment of the particle size distribution offers valuable information on the powder flowing and compression behaviors, and also it influences the tablets’ uniformity of content and disintegration performance [37].

**Figure 13:**
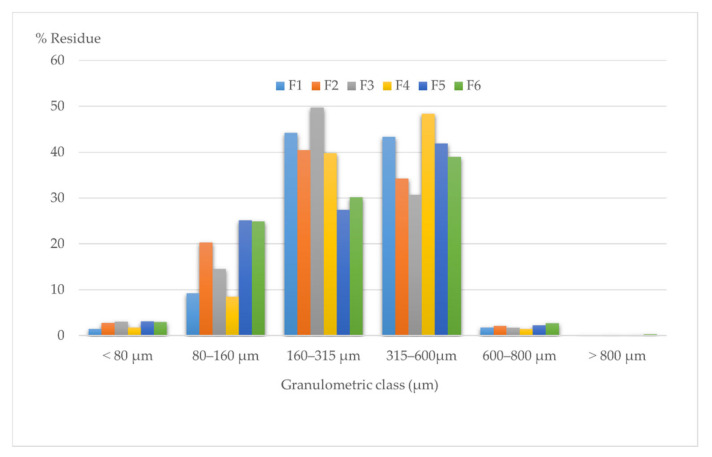
Granulometric analysis of the direct compression blends.

Figure 13 presents the histogram registered by representing the particle size distribution on granulometric classes for all six formulations of powders for direct compression.

The histogram plot displayed the differences in particle sizes between the formulations. The distribution of the various formulations follows heterodispersed features. All six formulations have a considerably broader particle size distribution, with most of them in the 80 to 600 µm range; those above 800 µm were almost not found. The registered results are typical for materials containing different components and it may tend to segregate those which will negatively influence the blend uniformity. Moreover, due to the difference in the particle sizes, each of the components can behave as individual particles [38,39].

F1 and F4 present similar characteristics, with the lowest variability, having a small part of the particles in ranges outside 160–600 µm, while F2, F3, F5, and F6 consist of particles with a wide variety of diameter ranges. However, a large fraction has finer particle PSDs in the 80–315 µm area, with no significant difference in habits between these four samples.

A more homogeneous distribution of the powder blend was achieved if particles exhibit a narrow particle size distribution. The particle size influences the other fundamental mechanical properties of the in-process material and the quality of the final tablets. Smaller particles, meaning a larger surface area, increase their solubility and dissolution rates, leading to a faster-acting drug release, but they tend to be more cohesive since the interparticle adhesion and frictional forces dominate, making them more compressible, leading to hard tablets with weak disintegration [40,41].

Flow behavior is an essential property for the accurate filling of the mold and is inherent for the uniform dosage of the tablets, while also being relevant for predicting manufacturing problems [42]. The volumetric characteristics provide helpful measures of the powder flowability and cohesivity [43]. 

Table 4 shows the moisture, flowability, and compressibility characteristics of the blend material.

Due to the amorphous character of the lyophilized powder used as an active ingredient, a certain moisture content was expected to be assessed. F3, F5, and F6 contain the highest amounts of water, over 8.5%, reaching 9.22% in F5, almost double 5.23% found in F1, demonstrating that the moisture degree does not depend on the type of cyclodextrin (HP-β-CD or Me-β-CD), or on the silicified microcrystalline cellulose used, but rather the F-MELT excipient added. F1 and F4 present the lowest content of humidity, with values suitable for the direct compression process. An increase in moisture content induces powder aggregation and leads to a lack of uniformity in filling the tablet machine die. The powder moisture directly affects the flowability, depending on the amount of water and its distribution.

In the case of the higher moisture content, the decrease in bonding strength exceeds the increase in the bonding area and results in a compressibility decrease [44]. This is the reason why F2, F3, F5, and F5 present a lower compressibility and flowability performance.

All six powders displayed a weak flowability, as it could be measured only when stirring with a speed of 10 rpm through a 15 mm nozzle. They did not steadily and consistently flow through a 10 mm nozzle, even when stirring, nor by a 15 mm nozzle at a lower speed rate. This behavior is characteristic for amorphous systems with low particle sizes. Still, the angle of the repose values obtained for F1 and F4 fall into the 25 to 30 range, considered by pharmacopoeia specifications as corresponding to an excellent flow [23]. Due to the experiment conditions, it cannot be stated that they show a great flowability, but it is suitable for the compression process, and it is better than the other four formulations. F5 presented the highest angle of repose value (39.42), closely followed by F6 and F3 (38.96 and 38.71, respectively), then F2 with 36.22, demonstrating an unsatisfying quality for these materials. 

The flowing time was around 12 s for F1 and F4 and above 13 s for the other formulations, proving the influence of the selected excipients on the compression blend. The highest flow time (over 17 s) and the lowest flow rate (3.428 g/s) were registered for F5, but similar values were also observed for F6, F2, and F3, certifying the weak flowing properties for these formulations. Although all used excipients are specially created for direct compression technology, as well as having great flowability and compressibility properties, the fact that the inclusion complex represents between 24 and 29% of the final formulations is directly affects the blend mechanical behavior. 

It is observed that the powders containing the Me-β-CD inclusion complex have diminished flowability attributes in comparison to the HP-β-CD inclusion complex powders, but also the incorporation of F-MELT in the formulation conducts a reduced flowing rate. 

The HR values confirmed the flowability results, with an obvious better flowing characteristic for F1 and F4 than the other four formulations. By the previously developed tests, F5 recorded the highest value of HR (1.44), followed by F2 (1.36), F3 (1.32), and F6 (1.29). The low bulk densities registered for F1 and F4 are specific for porous structures which facilitate the compressibility, as it appears from CI values. In terms of CI, there are significant differences between formulations, proving that a density decrease improves the tableting properties. The calculating values of CI confirm the HR results, with F5 also presenting the highest value, maintaining the same order for the compressibility performance as for flowability. It is obvious that the density (bulk or tapped) increases with the decreasing particle size. Wider particle-size distribution by compression mixtures induced a denser packing of the particles. In a wide particle-size distribution, different particle rearrangements can be expected, in which smaller particles fill the gaps between larger particles, resulting in denser material [45].

It can be remarked that the type of cyclodextrin does not influence the volumetric blend attributes, the excipients play an important role. The results prove that the use of F-MELT in the powder formulations lead to a notable decrease in the essential compression properties, compared to the association between silicified microcrystalline cellulose and DISINTEQUIK.

The deformation mechanism and the physical and chemical properties of the blends impose on their performance. 

Based on the obtained results for fundamental mechanical properties, F1 and F4 display the best plasticity due to the relatively low density, a low moisture content preferable for the direct compression process, and a good flowability justified by the uniformity of the particle size distribution, which are the reasons why we selected these two formulations for the following studies.

*Quality characterization of the orodispersible tablets*

*Organoleptic evaluation*

The appearance and color uniformity provide the first evidence of a correct formulation and manufacturing of the tablets, and they are essential for patient compliance.

**Figure 14:**
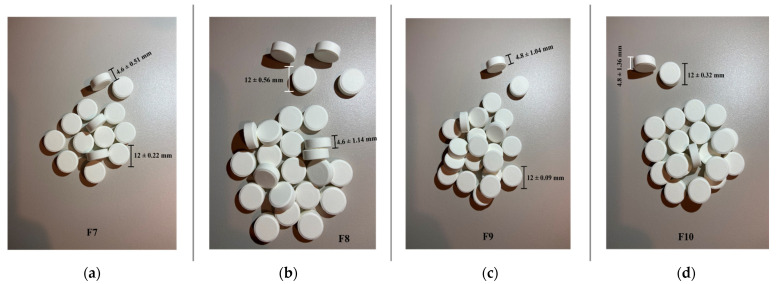
The general appearance of the four tablet formulations (**a**)—F7, (**b**)—F8, (**c**)—F9, (**d**)—F10.

All four formulations lead to round-shaped tablets with smooth flat surfaces and uniform appearances, with no capping or lamination observed, colored in white (Figure 14).

The uniformity of the tablet sizes offers information on the compression process, as it is well known that thickness may vary, with no weight change, due to the material difference in density or the applied force [46]. The mass uniformity represents an indicator for a well-conducted compression process and for an accurate dosage of the tablets [47].

Hardness is a measure of the tablet crushing strength and is directly influenced by the disintegration time, which is the most important parameter for orodispersible tablets, as well as their resistance to breakage under mechanical shaking before usage. The tablet breaking force highly depends on the uniformity of the in-process material and on the compression force. The orally disintegrating tablets are preferably having a lower hardness than the conventional tablets, as an increased one will delay the disintegration [48,49]. 

Another analysis that measures the strength of the tablets is the friability test. Friability is the tablets’ physical properties which show their behaviors in time under mechanical stress during handling before administration [50].

Orodispersible tablets are designed to disintegrate or dissolve rapidly on contact with saliva, and this is the most significant difference from all the other types of tablets [51]. Considering this, the most important characteristic of the developed formulations is represented by the disintegration tablets.

The pharmacotechnical and in vitro evaluation results are shown in Table 5.

From Table 5, it can be observed there are no significant differences in the results between the studied formulations, leading to the conclusion that the type of used cyclodextrins used, or the various compression forces, have no critical influence on the orally disintegrating tablets.

Still, some distinctions can be detected between the batches.

Concerning the tablet sizes, all formulations have an average 12 mm diameter, with a deviation that does not exceed ± 1%. The uniformity of sizes follows the pharmacopoeial standard. The slight deviations in tablets’ diameters are either due to an uneven feeding of powder in the die or to the irregular movement of the lower punch. In the case of tablets for oral dispersion, the sizes can affect the disintegration time and, by this, the therapeutic efficacy [52] The registered size uniformity for the studied tablets confirms the good pharmacotechnical properties of the powder blend and the well-established compression parameters.

Regarding the tablet’s thickness, there was a slight difference between batches depending on the type of cyclodextrin used in the inclusion complex; thus, for HP-β-CD, the height is lower than for Me-β-CD, somehow producing a predictable result considering the CI values which were higher for the Me-β-CD material, and the HP-β-CD blend presented a better compressibility in the specific test.

All the tablet’s mass uniformities met the requirements of the European Pharmacopoeia (±5%) [23]. The mass variation depends more on the direct compression mixture characteristics, such as the particle size distribution, flowability, and compressibility, and less on the compression force or procedure, which are the direct results of the suitable tablet composition. The die-fill uniformity is a limiting factor for the direct compression method, and the weight uniformity between the tablets of the same batch or different batches, with low SD values, proves the correct formulation selection [53].

Concerning the hardness of the tablets, the impact of the compression force is obvious. It can be easily observed that F8 and F10, manufactured at high compression force, are presenting an increased hardness. As no other factor was changed, in composition or in process, the influence of the tableting force is significant. For tablets containing Nim–HP-β-CD inclusion complex, the mechanical resistance was 40.2 N for 30 N compression force and 53.9 N for 60 N force. Similar values are obtained for tablets containing Nim–Me-β-CD inclusion complex. Considering that the hardness is more than ensuring the mechanical integrity, as it is directly related to all other pharmacotechnical attributes, important information on the differences in disintegration time were offered [54].

Even the friability test is closely related to the tablet hardness, and in terms of friability, the results are similar between the four formulations, in comparison with hardness values which are remarkably different, a function of the applied force. All batches of orally disintegrating tablets are displaying an ideal friability and stability, hard to be obtained for this type of dosage form, and in compliance with approved regulations. The friability is totally depending on the formulation and direct compression mixture physical properties.

The disintegration time for all four formulations present ideal values for orally disintegrating tablets, strengthening the idea that the selection of types and amounts of excipients were accurate. Some differences were registered, but they seem to depend more on the applied compression force than the type of cyclodextrin used. Predictably, considering the hardness results, the disintegration time was longer for tablets manufactured at a high force, but they still comply with the regulations. 

Managing to maintain the balance between hardness, friability, and disintegration properties is a real success of the formulation and manufacturing process, taking into account that disintegration time is the orodispersible tablets’ critical quality attribute.

Moreover, it was not proven that there was a significant difference in the disintegration time in the two utilized media, but we noticed that for simulated saliva, the values are a little higher. The satisfactory disintegration is due to the pair of superdisintegrants, DISINTEQUIK™ ODT and EXPLOTAB^®^, chosen in the formulation studies.

However, as the fast disintegration of the orally disintegrating tablets does not always undergo rapid dissolution, establishing the dissolution rate in different media is very important [55].

Regarding the dissolution results, it can also be noticed that the release rate has similar values for all four formulations and is not dependent on the compression force, on the cyclodextrin type, or on the dissolution medium nature. The obtained values meet the pharmacopoeia standards, and after 30 min the nimodipine release is almost complete. The dissolution was rapid with a low inter-batch variability, as well as between the individual profile’s variability [56]. Regardless of the dissolution media or type of formulation, the dissolution rate registered values above 95% after 30 min, for all studied batches. This performance is due to both the inclusion process of nimodipine in the cyclodextrin cavity and the selected excipients. It is well-known that nimodipine has a hydrophobic character which causes its floating at the surface of the dissolution medium [57], but this behavior was not identified in the present study when it was encapsulated in cyclodextrins.

## 4. Conclusions

In this research, inclusion complexes of nimodipine with two different cyclodextrins, namely HP-β-CD and Me-β-CD, were prepared using three different working procedures. The obtained inclusion complexes, in a 1:1 molar ratio, were evaluated in comparison with a physical mixture with the same quantity. The FTIR, SEM, DSC, and XRD analyses confirmed that the inclusion was successful when the lyophilization procedure was applied. Therefore, new orally disintegrating tablets containing nimodipine–hydroxypropyl-β-cyclodextrin and nimodipine–methyl-β-cyclodextrin inclusion complexes were developed. The proposed research established if HP-β-CD and Me-β-CD can include nimodipine in their cavity, as well as which method of complex preparation (lyophilization, coprecipitation, and kneading techniques) was more suitable, and the best formulation and manufacturing parameters for obtaining orally disintegrating tablets with inclusion complexes in order to develop tablets which meet the compendial specifications. This was successfully reached. 

In conclusion, the type of cyclodextrin does not influence the physical and in vitro properties of the manufactured orally disintegrating tablets. Both of them ensure desirable quality characteristics. The lyophilization method proved to be more effective to obtaining an inclusion complex in a 1:1 molar ratio for both HP-β-CD and Me-β-CD compounds. 

Our future study is oriented on registering the dissolution profiles, in various media, of orodispersible tablets containing nimodipine inclusion complexes in cyclodextrins and analyzing the release kinetics.

## Figures and Tables

**Table 1:** The formulations of the direct compression mixture with Nim–HP-β-CD and Nim–Me-β-CD inclusion complexes.

Ingredients	Formulation/Amount (%)					
	F1	F2	F3	F4	F5	F6
Nim–HP-β-CD	28.10	28.10	28.10	-	-	-
Nim–Me-β-CD	-	-	-	24.92	24.92	24.92
PROSOLV^®^ SMCC HD 90—Silicified microcrystalline cellulose	58.90	-	35.00	62.08	-	36.00
F-MELT^®^	-	65.90	35.90	-	69.08	38.08
DISINTEQUIK™ ODT	10.00	-	-	10.00	-	-
EXPLOTAB^®^—Sodium starch glycolate	2.00	5.00	-	2.00	5.00	-
LIGAMED^®^ MF-2-V—Magnesium stearate	1.00	1.00	1.00	1.00	1.00	1.00

**Table 2:** The formulations of the tablets with Nim–HP-β-CD and Nim–Me-β-CD inclusion complexes.

Ingredients	Quantity mg/Tablet				Role in Formulation
	F7 (F1—30 N)	F8 (F1—60 N)	F9 (F1—30 N)	F10 (F1—60 N)	
Inclusion complex Nim–HP-β-CD (1:1)	140.50	140.50	-	-	Active ingredient
Inclusion complex Nim–Me-β-CD (1:1)	-	-	124.60	124.60	Active ingredient
PROSOLV^®^ SMCC HD 90—Silicified microcrystalline cellulose	294.50	294.50	310.40	310.40	Filler Binder
DISINTEQUIK™ ODT	50.00	50.00	50.00	50.00	Superdisintegrant
EXPLOTAB^®^—Sodium starch glycolate	10.00	10.00	10.00	10.00	Superdisintegrant
LIGAMED^®^ MF-2-V—Magnesium stearate	5.00	5.00	5.00	5.00	Lubricant
TOTAL	500.00	500.00	500.00	500.00	

**Table 3:** Thermodynamic parameters for process of samples from DSC measurements.

Sample	*T*_onset_/°C	*T*_max_/°C	*T*_end_/°C	Δ*H/*(J g^−1^)
Nimodipine	124.53	126.48	127.89	39.1
HP-β-CD	208.55	209.22	210.92	43.14
Nim–HP-β-CD physical mixture	116.29	118.52	120.32	12.66
Nim–HP-β-CD coprecipitation	114.32	118.22	120.25	103.8
	123.88	124.37	125.02	0.6
Nim–HP-β-CD lyophilization	107.38	108.83	111.41	9.6
Nim–HP-β-CD kneading	116.22	118.3	120.01	7.72
	199.62	206.7	212.5	20.93
	282.03	282.49	283.26	16.72
	283.85	285.83	287.05	23.79
Me-β-CD	209.9	210.33	211.1	0.193
	285.53	286.0	286.77	13.97
	287.27	289.08	294.99	21.75
Nim–Me-β-CD physical mixture	115.74	119.74	122.19	21.47
	225.56	226.07	226.65	1.28
	282.03	284.71	283.85	2.4
	283.9	291.52	291.36	24.99
	336.05	341.72	349.99	16.77
Nim–Me-β-CD coprecipitation	114.51	115.67	116.88	95.18
Nim–Me-β-CD lyophilization	120.8	125.57	126.19	5.66
	337.14	342.05	345.52	5.74
Nim–Me-β-CD kneading	113.5	118.8	120.04	5.61
	229.64	229.97	230.75	3.86
	286.53	286.63	287.3	13.06
	287.65	291.54	326.38	83.28

**Table 4:** Precompression parameters for the direct compression blends.

Parameter	Formulation Code					
	F1	F2	F3	F4	F5	F6
Moisture content (%)	5.23 ± 1.17	7.98 ± 2.12	8.64 ± 1.28	5.77 ± 1.66	9.22 ± 2.45	8.56 ± 2.74
Flow time (s) *	12.4 ± 2.75	13.6 ± 3.87	14.8 ± 2.91	11.7 ± 2.34	17.5 ± 3.12	15.9 ± 3.44
Angle of repose (θ degrees) *	30.16 ± 2.19	36.22 ± 3.14	38.71 ± 3.05	29.12 ± 1.77	39.42 ± 3.86	38.96 ± 3.52
Flow rate (g/s) *	4.838	4.411	4.054	5.128	3.428	3.773
Bulk density (g/mL)	0.318 ± 0.10	0.372 ± 0.09	0.388 ± 0.10	0.306 ± 0.05	0.403 ± 0.12	0.397 ± 0.10
Tapped density (g/mL)	0.375 ± 0.08	0.508 ± 0.65	0.513 ± 0.46	0.376 ± 0.22	0.581 ± 0.43	0.516 ± 0.63
Carr Index (CI) (%)	15.2	26.77	24.36	18.61	30.63	23.06
Hausner’s ratio (HR)	1.17	1.36	1.32	1.22	1.44	1.29

* stirring speed: 10 rpm; nozzle: 15 mm.

**Table 5:** Quality properties of the studied tablets.

Tested Parameters	Formulation Code			
	F7	F8	F9	F10
Thickness (mm)	4.6 ± 0.51	4.6 ± 1.14	4.8 ± 1.04	4.8 ± 1.36
Diameter (mm)	12 ± 0.22	12 ± 0.56	12 ± 0.09	12 ± 0.32
Mass uniformity	497 ± 2.75	495 ± 3.51	498 ± 2.18	492 ± 5.43
Hardness (N)	40.2 ± 3.58	53.9 ± 4.72	43.6 ± 2.97	55.6 ± 3.24
Friability (%)	0.12 ± 0.18	0.16 ± 0.23	0.11 ± 0.45	0.26 ± 0.59
In vitro disintegration time—in water medium (seconds)	28 ± 1.88	30 ± 2.17	29 ± 2.46	36 ± 2.95
In vitro disintegration time—in simulated saliva medium (seconds)	32 ± 2.21	35 ± 3.14	34 ± 2.74	42 ± 3.61
In vitro dissolution rate—in acidic medium, after 30 min (%)	96.85 ± 1.92	95.77 ± 2.15	97.04 ± 1.63	96.28 ± 1.59
In vitro disintegration time—in simulated saliva medium, after 30 min (%)	97.21 ± 1.05	96.35 ± 1.22	97.43 ± 1.18	97.16 ± 2.36

## Data Availability

Not applicable.

## Sample Availability

Samples of the compounds are available from the authors.
